# Exploring the Mechanism of Alyteserin‐1c in Gram‐Positive and Gram‐Negative Bacterial Membrane Models Using a Computational Approach

**DOI:** 10.1002/mbo3.70327

**Published:** 2026-06-10

**Authors:** Akshay Sasidharan, Rajasekaran Ramalingam

**Affiliations:** ^1^ Quantitative Biology Lab, Department of Integrative Biology, School of Bio Sciences and Technology Vellore Institute of Technology (VIT Deemed to be University) Vellore Tamil Nadu India

**Keywords:** Alyteserin‐1c, gram‐negative membrane, gram‐positive membrane, lipid bilayer selectivity, membrane disruption mechanism, molecular dynamics simulation

## Abstract

Alyteserin‐1c is a 23‐amino‐acid cationic antimicrobial peptide exhibiting greater activity against Gram‐negative than Gram‐positive bacteria (Conlon et al. 2009), yet the molecular basis of this differential membrane selectivity remains incompletely understood. Using all‐atom molecular dynamics simulations (200 ns × 3 independent replicates per system), we examined the interactions of a pre‐assembled Alyteserin‐1c hexamer with simplified bilayer models representing Gram‐positive (75% PG/25% PE) and Gram‐negative (75% PE/25% PG) bacterial inner membranes. The hexameric assembly maintained structural stability and substantial α‐helical content throughout all simulations, with oligomeric cohesion associated primarily with hydrophobic packing of residues L10, L13, V14, I17, and F6. Cationic residues K7 and K15 remained lipid‐exposed with near‐permanent headgroup contact occupancy, suggesting their role as principal membrane‐anchoring sites. Comparative membrane analyses indicated greater lateral lipid mobility, lower bilayer density, and stronger local acyl chain perturbation in the Gram‐negative membrane model, consistent with greater membrane susceptibility at lower peptide concentrations. Helix orientation and pore geometry analyses were inconsistent with stable barrel‐stave transmembrane pore formation and instead supported interfacial or transient toroidal‐like membrane perturbations. Transient single‐file water threading was observed in the Gram‐positive system, while perturbations in the Gram‐negative membrane remained predominantly surface‐localized. These findings provide a computationally derived mechanistic framework for the membrane selectivity of Alyteserin‐1c and may inform rational design of membrane‐targeting antimicrobial peptides. Given the simplified bilayer models, pre‐assembled oligomeric assumption, and accessible simulation timescales, the proposed mechanisms should be interpreted as hypothesis‐generating rather than definitive.

## Graphical abstract

Alyteserin‐1c hexamer was investigated in biomimetic Gram‐positive and Gram‐negative membrane models using all‐atom molecular dynamics simulations to understand its membrane disruption behavior. The hexamer remained structurally stable in both membrane environments, with hydrophobic inter‐monomer interactions maintaining oligomer integrity while cationic residues mediated strong electrostatic lipid interactions. Distinct membrane‐dependent responses were observed, with the Gram‐negative membrane exhibiting greater lipid mobility and stronger peptide–membrane electrostatic interactions. Collectively, these findings support a predominantly interfacial carpet/toroidal‐like membrane disruption mechanism for Alyteserin‐1c.

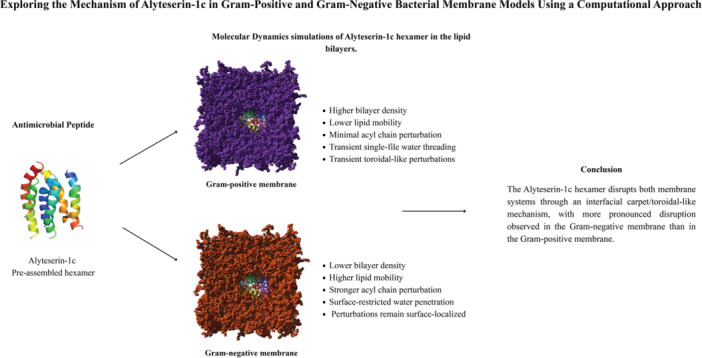

**Abbreviations**
AMP: antimicrobial peptide
AMR: antimicrobial resistance
DSSP: define secondary structure of proteins
Dxy: lateral diffusion coefficient
ff14SB: AMBER Force Field 14 Sidechain and Backbone
GN: gram‐negative membrane model
GP: gram‐positive membrane model
LIPID17: AMBER Force Field for Lipid Simulations (2017 version)
MD: molecular dynamics
MSD: mean square displacement
OL: oleoyl acyl chain (AMBER LIPID17)
PA: palmitoyl acyl chain (AMBER LIPID17)
PE: phosphatidylethanolamine
PG: phosphatidylglycerol
PGR: phosphatidylglycerol headgroup residue (AMBER LIPID17)
Rg: radius of gyration
RMSD: root mean square deviation
SCD: segmental deuterium order parameter (lipid order parameter)
SEM: standard error of the mean
VMD: visual molecular dynamics

## 1 Introduction

The growing prevalence of antimicrobial resistance (AMR) has impeded the efficacy of current antibiotics and necessitates the development of novel antimicrobial agents with distinct mechanisms of action (Magana et al. 2020). One such potential antimicrobial agent is antimicrobial peptides (AMPs). AMPs can target organisms ranging from viruses to parasites and their mode of action is different from those of traditional antibiotics (Aragón‐Muriel et al. 2019). AMPs usually exhibit low biological toxicity and exert membranolytic action on bacterial cell membranes without any specific receptors (Hollmann et al. 2018; Moravej et al. 2018). Among naturally occurring antimicrobial peptides (AMPs), α‐helical cationic peptides derived from amphibian skin secretions have attracted significant attention because of their broad‐spectrum antibacterial activity, minimal toxicity toward host cells, and their selective ability to target bacterial membranes over mammalian membranes (Moravej et al. 2018). Amphibians at various taxonomic levels possess distinct repertoires of antimicrobial peptides, each associated with different AMP families (Canè et al. 2024). Examples of antimicrobial peptides derived from amphibian skin include brevinins, esculentins, ranacyclins, temporins, magainins, dermaseptins, and alyteserins (Ladram 2016).

Antibacterial AMPs represent the extensively investigated class of antimicrobial peptides (Rivas et al. 2009). Most AMPs are cationic and amphipathic, that promote effective interaction with bacterial membranes, resulting in disruption of the lipid bilayer structure (Jenssen et al. 2006). AMPs interact with membranes through various mechanisms. In the carpet model, peptides cover and disrupt the membrane surface. Membrane thinning involves the insertion of AMPs into one leaflet that causes lipid displacement. Aggregate formation involves clustering of AMPs into sphere‐like structures within the membrane. In toroidal pore formation, peptides induce membrane curvature to form continuous, lipid‐lined pores. Conversely in barrel‐stave model, peptides insert perpendicularly to form transmembrane channels (Bahar and Ren 2013). The selection of mechanism depends on multiple factors including peptide concentration, lipid composition and membrane physical properties (fluidity, thickness and charge) (Talapko et al. 2022). Distinguishing among these mechanisms is critical for rational peptide design, as each model implies different structure‐activity relationships and lipid composition dependencies (Chen et al. 2021). Many AMPs, including peptides from the alyteserin family, display Gram‐type selectivity with 2‐4‐fold differences in minimum inhibitory concentrations (MIC) between bacterial classes (Conlon et al. 2009). However, the molecular mechanism of this selectivity, whether it is arising from differential binding affinities, distinct pore architectures, or variations in membrane disruption kinetics, remains poorly understood due to limitations of experimental techniques in resolving transient pore structures at atomic resolution (Berkowitz et al. 1990).

Alyteserin‐1c is a 23‐amino‐acid (sequence: GLKEIFKAGLGSLVKGIAAHVAS), with a net charge of +2 (cationic), α‐helical peptide initially isolated from norepinephrine‐stimulated skin secretions of the midwife toad *Alytes obstetricans*. Studies on Alyteserin‐1c have shown selective inhibitory activity against *Escherichia coli* (Gram‐negative: Minimum Inhibitory Concentration, MIC = 25 μM) and *Staphylococcus aureus* (Gram‐positive: Minimum Inhibitory Concentration, MIC = 100 μM), along with low hemolytic activity against human erythrocytes (Lethal concentration 50%, LC_50_ = 145 μM) (Conlon et al. 2009). Previous molecular dynamics (MD) simulations of monomeric Alyteserin‐1c and its cationic (+5) analog have focused on simplified POPC/POPG vesicles in the ratio of 3:1 mimicking a gram‐negative membrane environment. The study was limited to 500 ps trajectories and identified surface adsorption and local lipid disordering but could not address the complete membrane disruption mechanism (Aragón‐Muriel et al. 2019). Monomeric simulations cannot elucidate cooperative oligomerization of peptides, which is a hallmark feature of pore‐forming AMPs, while some experimental evidence from other α‐helical peptides (magainins, alamethicin, protegrins) demonstrates that membrane pore formation requires oligomeric assemblies, typically tetramers to octamers, yet the oligomeric state and pore architecture of Alyteserin‐1c remain uncharacterized (Brogden 2005; Wimley and Hristova 2020).

In our study, we employed all‐atom molecular dynamics simulations (200 ns per system, *n* = 3 independent replicates) to investigate the membrane disruption mechanism of pre‐assembled Alyteserin‐1c hexamer in biomimetic inner membranes representing Gram‐positive and Gram‐negative bacteria. The Gram‐positive inner membrane model (GP) consisted of 25% phosphatidylethanolamine (PE) and 75% phosphatidylglycerol (PG), while the Gram‐negative inner membrane model (GN) contained an inverted ratio of 75% phosphatidylethanolamine (PE) and 25% phosphatidylglycerol (PG) to represent a less anionic, zwitterionic‐dominant surface (Pogozheva et al. 2022). Our primary aim was to identify molecular determinants of membrane selectivity and characterize the mechanism of membrane disruption at atomistic resolution. By integrating analyses of peptide oligomer stability, inter‐monomer cohesion, membrane structural perturbations, pore geometry and hydration, system energetics, and lipid acyl chain ordering, we provide a computationally derived mechanistic framework underlying the membrane selectivity of Alyteserin‐1c and may facilitate the rational design of membrane‐targeting antimicrobial peptides.

## 2 Methodology

### 2.1 Peptide Structure Retrieval and Refinement

Initially, the three‐dimensional structure of Alyteserin‐1c peptide was retrieved from the Protein Data Bank (PDB) database with PDB ID: 2L5R. The peptide structure was subjected to energy minimization using AMBER 14 force field to reduce the potential energy, relieve steric clashes, and obtained a more stable conformation (Maier et al. 2015).

### 2.2 Oligomerization of Peptides

Oligomeric assemblies of Alyteserin‐1c were constructed using the ClusPro 2.0 platform, which employs the PIPER protein–protein docking algorithm (Kozakov et al. 2017). The monomeric peptide structure was initially used as both receptor and ligand to generate dimeric complexes. The resulting dimer was then sequentially docked with additional monomeric units to facilitate the stepwise construction of higher‐order non‐covalently associated oligomers, including trimers, tetramers, pentamers, hexamers, and heptamers. The PIPER algorithm performs rigid‐body docking by sampling a large ensemble of candidate conformations, clustering low‐energy poses, and ranking them using scoring functions that incorporate electrostatic, hydrophobic, and van der Waals interaction terms (Kozakov et al. 2006). In this study, the default “Balanced” scoring mode was applied, which combines electrostatic interactions, van der Waals forces, and desolvation energy contributions for model evaluation and ranking. At each stage of oligomerization, the top‐ranked docking model was selected for subsequent analyses and used as the structural template for generating the next oligomeric assembly.

### 2.3 Protein Flexibility Analysis

Subsequently, the conformational flexibility and mechanical stability of the Alyteserin‐1c oligomers were evaluated using ProFlex, a constraint network‐based computational framework. In this approach, covalent bonds, hydrogen bonds, and hydrophobic interactions were represented as mechanical constraints, whereas rotatable bonds were treated as degrees of freedom (Jacobs et al. 2001). ProFlex characterizes the intrinsic rigidity of a structure through a hydrogen bond dilution procedure, in which hydrogen bonds are progressively removed in order of decreasing strength. This analysis enables the identification of rigid clusters, flexible regions, and hinge points within the oligomeric assemblies, thereby providing insight into their mechanical stability (Wells et al. 2005). All oligomeric models generated using ClusPro 2.0 were subjected to rigidity analysis through Linux command‐line execution of ProFlex. The initial hydrogen bond energy cutoff was set to −1.0 kcal·mol^−1^, and dilution was carried out by systematically lowering this threshold to mimic the progressive weakening of non‐covalent interactions. The resulting rigidity profiles were compared across oligomeric states ranging from monomer to hexamer to identify the assembly exhibiting the most suitable balance between structural rigidity and conformational flexibility for subsequent membrane simulation studies.

### 2.4 Molecular Dynamics (MD) Simulation

All‐atom MD simulations were carried out using GROMACS 2024.4 with the AMBER ff14SB force field for the peptide and AMBER LIPID17 parameters for membrane lipids (Maier et al. 2015; Abraham et al. 2015; Gould et al. 2018). Each peptide‐membrane system was simulated in triplicate (*n* = 3 independent replicates per membrane type) for 200 ns. To ensure statistical independence among simulations, each replicate was initiated using a distinct random seed for velocity generation. Two lipid bilayer models representing the characteristic inner membrane compositions of Gram‐positive and Gram‐negative bacteria were constructed using the CHARMM‐GUI Membrane Builder (Jo et al. 2008; Wu 2014). The Gram‐positive membrane model comprised 75% phosphatidylglycerol (PG) and 25% phosphatidylethanolamine (PE), reflecting the highly anionic nature of Gram‐positive cytoplasmic membranes. In contrast, the Gram‐negative membrane model employed an inverted composition of 75% PE and 25% PG, representing the comparatively less anionic and zwitterionic‐dominant inner membrane characteristic of Gram‐negative bacteria (Pogozheva et al. 2022). In both membrane systems, PE lipids were represented using palmitoyl (PA) and oleoyl (OL) acyl chain residues within the AMBER LIPID17 force field according to the split‐residue convention, whereas PG lipids were represented using the PGR headgroup residue. The membrane models used in this study were designed as simplified biomimetic representations of bacterial inner membrane lipid compositions rather than complete reconstructions of native bacterial envelopes. For the Gram‐negative system, the model represents the cytoplasmic (inner) membrane and does not include the outer membrane components, such as lipopolysaccharides (LPS), that characterize the asymmetric double‐membrane architecture of Gram‐negative bacteria (Paulowski et al. 2020). Likewise, the Gram‐positive membrane model does not incorporate structural features of the cell wall, including the peptidoglycan layer and teichoic acids. Despite these simplifications, PE/PG binary bilayer systems are widely employed in antimicrobial peptide–membrane molecular dynamics simulations because they provide a computationally tractable framework for systematically investigating membrane composition–dependent interactions and disruption mechanisms (Pogozheva et al. 2022; Paulowski et al. 2020)

The pre‐assembled Alyteserin‐1c oligomer generated via rigid‐body docking (ClusPro) were oriented in the membrane using the OPM server (Orientation of Proteins in Membranes) (PPM 3.0) (Lomize et al. 2012). The oligomer was inserted into the bilayer center using the CHARMM‐GUI peptide insertion protocol. The ends of each monomer were treated in accordance with the native peptide sequence. The N‐terminus of Glycine‐1 was maintained as a free amino group (NGLY in AMBER nomenclature), and the C‐terminus of Serine‐23 was amidated (CSER), which is consistent with the experimentally characterized C‐terminal amide of Alyteserin‐1c (Conlon et al. 2009). The capping accurately reproduces the net charge and terminal chemistry of the native peptide. Each system was solvated with explicit TIP3P water molecules in a periodic rectangular simulation box and neutralized with Na^+^ and Cl^−^ ions at a physiological concentration of 150 mM. Energy minimization was performed using the steepest descent algorithm until the maximum force converged to an acceptable threshold. The minimized system was subsequently subjected to a six‐stage equilibration protocol generated by CHARMM‐GUI, in which positional restraints on the peptide backbone, lipid headgroups, and acyl chains were progressively reduced across successive NPT stages.

Production simulations were performed under the NPT ensemble at 310 K and 1.0 bar for 200 ns per replicate (integration timestep of 2 fs). Temperature was maintained at 310 K using the Nosé‐Hoover thermostat applied separately to the solute‐membrane and solvent groups (Nosé 1984). Pressure was maintained at 1.0 bar using the Parrinello‐Rahman barostat with semi‐isotropic coupling to allow the bilayer XY plane and *Z*‐axis to scale independently (Parrinello and Rahman 1981). Long‐range electrostatic interactions were computed using the Particle Mesh Ewald (PME) method with a 1.2 nm cutoff, and van der Waals interactions were treated with a force‐switch modifier decaying to zero at 1.2 nm (Darden et al. 1993). All covalent bonds involving hydrogen atoms were constrained using the LINCS algorithm (Hess et al. 1997). Center‐of‐mass motion was removed linearly every 100 steps to prevent artificial drift. Post‐processing of trajectories (xtc) was performed using GROMACS trjconv utilities. To reduce computational cost while maintaining representative sampling, trajectory frames were sampled using the ‐skip 10 option in gmx, reducing the dataset from 2001 frames to 201 frames for subsequent analysis. For membrane‐specific analyses requiring intact bilayer representation, trajectories were re‐centered on the protein in the XY plane with periodic boundary condition (PBC) correction (Yeh and Hummer 2004). All the analyses were conducted on production trajectories using GROMACS built‐in tools (gmx tools) and custom Python scripts employing MDAnalysis (v2.9.0) (Michaud‐Agrawal et al. 2011; Gowers 2019).

### 2.5 System Energetics

System‐wide energetic properties were calculated from the GROMACS energy output files using the command gmx energy. The following characteristics were assessed throughout each production trajectory: Lennard‐Jones short‐range interaction energy (LJ‐SR), Coulomb short‐range interaction energy (Coulomb‐SR), and total potential energy. Temperature and pressure were also recorded to confirm the thermodynamic equilibration of each system. Because the Gram‐positive and Gram‐negative systems differed in total atom count, absolute energy values could not be directly compared between the two systems. Therefore, simulation convergence was evaluated primarily based on the temporal stability of individual energy terms, while the Coulomb‐SR component was specifically interpreted in the context of electrostatic interactions between the cationic peptide oligomer and anionic membrane lipids. All energy parameters are reported as mean ± SEM obtained from three independent simulation replicates.

### 2.6 Structural Stability

The structural integrity and conformational stability of the Alyteserin‐1c oligomer were assessed by computing all 201 production trajectory frames. Backbone root‐mean‐square deviation (RMSD) was calculated using gmx rms with respect to the energy‐minimized starting structure, selecting all Cα atoms of the oligomer. The radius of gyration Rg was computed using gmx gyrate to monitor the compactness of the oligomeric assembly over time, with a decrease or stable plateau indicating maintained bundle integrity. Secondary structure content was calculated using the DSSP algorithm as implemented in MDAnalysis, and the percentage of residues adopting an α‐helical conformation was tracked throughout the trajectory (Nosé 1984). All three metrics were computed for each replicate independently and are reported as mean ± SEM across three replicates.

### 2.7 Inter‐Monomer Stability and Peptide–Membrane Interaction Analysis

To elucidate the molecular basis of oligomer stability, inter‐monomer interactions were calculated using three complementary analyses. First, the inter‐monomer hydrogen bonds were identified using a distance‐based criterion (donor–acceptor distance < 3.5 Å), counting N–O contacts between backbone and sidechain atoms belonging to different monomers at each trajectory frame. Second, inter‐monomer salt bridges were identified as contacts between cationic nitrogen atoms (LYS NZ) and anionic oxygen atoms (GLU OE1/OE2) of different monomers within a cutoff distance of 4.0 Å. Third, a monomer–monomer contact map was constructed for the M1–M2 interface as a representative adjacent monomer pair, by computing the frequency of Cα–Cα distances below 8.0 Å between all 23 residue positions of the two monomers, sampled every 10 frames. The M1–M2 pair was selected as representative because the hexameric assembly generated by ClusPro adopts an approximately symmetric bundle geometry in which adjacent monomer pairs share equivalent interfacial contacts. This was further supported by the inter‐monomer hydrogen bond analysis, which identified the M4–M5 pair (GN) and M1–M6 pair (GP) as the most active pairs, both involving adjacent monomers and confirming that the contact pattern observed at M1–M2 is characteristic of the hexameric interface rather than a pair‐specific artifact. All inter‐monomer analyses were implemented using custom Python scripts with the MDAnalysis package and are reported as mean ± SEM across three independent replicates (Michaud‐Agrawal et al. 2011; Gowers 2019).

Interactions between the Alyteserin‐1c oligomer and membrane lipids were analyzed through hydrogen bond analysis and residue‐level contact occupancy. Peptide–lipid hydrogen bonds were computed using gmx hbond with default geometric criteria (donor–acceptor distance < 3.5 Å, donor–hydrogen–acceptor angle > 150°), selecting the peptide as the donor/acceptor and all lipid molecules as the partner group. Peptide–water hydrogen bonds were calculated equivalently using TIP3P water molecules to monitor changes in peptide hydration upon membrane insertion. Residue‐level peptide–lipid contact occupancy was calculated for each residue position as the fraction of trajectory frames in which at least one lipid heavy atom was within 4.5 Å of any atom of that residue, averaged across all six monomers and expressed as a percentage. Residues with an occupancy ≥ 75% were classified as high‐contact anchoring residues, and residues with an occupancy between 50% and 75% were classified as medium‐contact residues. All the analysis were implemented using Python scripts using MDAnalysis package and calculations were performed per replicate and are reported as mean ± SEM across three replicates.

### 2.8 Membrane Property Analysis

Membrane structural perturbations induced by the Alyteserin‐1c oligomer were analyzed through a comprehensive set of analyses including lipid order parameters, lipid tilt angles, bilayer thickness, and membrane density profiles. All membrane analyses were performed on the production trajectories using a combination of GROMACS built‐in tools, MEMBPLUGIN 1.1 in VMD, and custom Python scripts employing the MDAnalysis library (v2.9.0) (Abraham et al. 2015; Gowers 2019; Guixà‐González et al. 2014; Humphrey et al. 1996). The mass density profile of membrane lipids along the bilayer normal (Z‐axis) was calculated using gmx density in GROMACS, selecting the total lipid group (PA, PE, OL, and PGR residues). Density profiles were calculated for each replicate and averaged to yield a mean profile. The peak‐to‐peak distance of the density profile was extracted as an independent, orthogonal estimate of bilayer thickness. The structural stability of the lipid bilayer was assessed by calculating the root‐mean‐square deviation (RMSD) and radius of gyration (*R*_*g*)_ of all lipid atoms over 200 ns using gmx rms and gmx gyrate, respectively. These metrics serve as baseline quality controls to confirm that the membrane remained structurally stable and did not undergo artificial macroscopic disruption during the simulation.

The segmental deuterium order parameter $S_{\left\{\text{CD}\right\}}$, also known as lipid order parameter, was calculated for the acyl chains of membrane lipids to quantify the orientational order and fluidity of the hydrocarbon core (Seelig and Seelig 1974). $S_{\left\{\text{CD}\right\}}$ is defined as $S_{\mathrm{CD}}=\frac{1}{2}\left(3\cos^{2}\theta -1\right)$ where θis the angle between each C─H bond vector and the bilayer normal (z‐axis). Calculations were performed separately for the sn‐1 palmitoyl chain (PA residues, C2–C16) and the sn‐2 oleoyl chain (OL residues, C2–C18. Hydrogen atoms were explicitly identified using the AMBER LIPID17 naming convention (HR/HS pairs for methylene carbons and H*T for terminal methyl groups). Values were averaged over all lipid molecules of each type and across all frames of each replicate. Results are reported as the absolute mean values across three independent replicates. Lipid tilt angles were calculated to assess lipid bilayer deformations induced by Alyteserin‐1c oligomer insertion. For phospholipid headgroup‐containing residues (PE and PGR), the tilt vector was defined from the phosphorus atom (P31) to the glycerol carbon (C1). For acyl chain residues (PA and OL), the tilt vector was defined along the chain axis from the terminal carbon (C116 or C118) to the glycerol‐ester carbon (C12). The tilt angle was calculated as the angle between each lipid vector and the normal membrane, and the absolute value of the cosine was used to treat upper and lower leaflets equivalently, resulting in tilt angles ranging from 0° to 90°. The bilayer thickness was calculated using MEMBPLUGIN 1.1, which computes the total membrane thickness as the distance between the mass‐density peaks of the phosphorus atoms (P31) in the upper and lower leaflets (Guixà‐González et al. 2014; Franco et al. 2022; Vinutha and Rajasekaran 2023). Trajectories were pre‐processed using gmx trjconv to center the system on the protein in the XY plane prior to analysis. Thickness maps were generated by averaging over all frames of each replicate, and mean bilayer thickness values were calculated from the resulting spatial distributions.

Finally, the lateral diffusion coefficient (Dxy) of membrane lipids was calculated from the mean square displacement (MSD) of individual lipid center‐of‐mass positions in the XY plane, using the Einstein relation for two‐dimensional diffusion:

Dxy=MSD(τ)/4τ,

where τ is the lag time (Michaud‐Agrawal et al. 2011). Center‐of‐mass positions were computed for each lipid residue (PA, PE, OL, and PGR) independently at every trajectory frame using MDAnalysis (v2.9.0) (Michaud‐Agrawal et al. 2011). To avoid the non‐equilibrium regime, MSD calculations were performed exclusively on the equilibrated portion of each trajectory (100–200 ns) (Koldsø et al. 2016). The MSD was computed using all available time origins within each replicate trajectory, and the linear diffusive regime (10%–40% of the maximum lag time) was identified and fitted using linear least‐squares regression. The slope of this fit was used to extract Dxy in units of Å^2^/ns, subsequently converted to cm^2^/s (1 Å^2^/ns = 1 × 10^−20^/10^−9^ m^2^/s). All calculations were implemented in custom Python scripts using MDAnalysis and NumPy, and Dxy values are reported as mean ± SEM across three independent replicates (Harris et al. 2020; Reback 2020).

### 2.9 Pore Radius Analysis

The geometry of the aqueous channel formed by the Alyteserin‐1c oligomer within each membrane was characterized using HOLE2, accessed through the MDAnalysis HoleAnalysis interface (v2.9.0) (Michaud‐Agrawal et al. 2011; Smart et al. 1996). HOLE2 calculates the pore radius along a defined axis by fitting the largest sphere that can pass through the protein interior at each position, thus providing a continuous radius profile as a function of positioning along the membrane‐normal (z‐axis). For each trajectory frame, a dynamic probe start point (cpoint) was defined using the median center‐of‐mass position of the oligomer in the XY plane, which is combined with the median bilayer center along the z‐axis (calculated from the mean z‐coordinates of phosphorus atoms (P31) of the upper and lower leaflets). The use of a median rather than a mean cpoint minimizes the bilayer drift and thermal fluctuations on probe placement. The probe was directed along the *z*‐axis (cvect = [0, 0, 1]) with a sampling resolution of 0.2 Å and a maximum search radius of 15.0 Å, beyond which the probe was considered to have existed into the bulk solvent.

Analyses were conducted on every fifth frame (step = 5) of each replicate trajectory. For each sampled frame, the minimum pore radius (*R*_min_) was extracted to identify the bottleneck of the channel. Frames in which the total z‐range of the HOLE2 profile was less than 35 Å were classified as “occluded,” indicating that the probe could not traverse the full bilayer thickness and that no continuous transmembrane channel was present. The non‐occluded frames were further classified as “closed” (*R*_min_ < 1.4 Å), narrow (R_min_ 1.4– R_min_ 2.8 Å), and open (*R*_min_ 2.8 Å) based on biophysically motivated thresholds. The value of 1.4 corresponds to the radius of a single water molecule, 2.8 Å to the minimum radius for unobstructed water passage, and 3.5 Å to the minimum radius for passage of a hydrated monovalent ion (Sunderland 2001). Mean pore radius profiles along the z‐axis were computed by interpolating individual frame profiles onto a common z‐grid using scipy. interpolate. interp1d and averaging across all non‐occluded frames within each replicate (Virtanen et al. 2020). Final values are reported as mean ± SEM across three independent replicates.

### 2.10 Hexamer Tilt Analysis

The orientation of individual Alyteserin‐1c monomers within the oligomeric assembly relative to the membrane‐normal was quantified by calculating the helix tilt angle for each of the six monomers independently across all production trajectory frames. All calculations were implemented using custom Python scripts employing MDAnalysis (v2.9.0), NumPy, and pandas (Michaud‐Agrawal et al. 2011; Harris et al. 2020; Reback 2020). For each monomer, the helix axis was defined using the principal axis of the Cα atoms spanning the core helical region Leu2–Val21 (residues 2–21 within each monomer. The principal axis was computed as the eigenvector associated with the largest moment of inertia of the selected Cα atoms, representing the longitudinal axis of the helix. At each trajectory frame, the hexamer was translated to the origin using its center of mass to prevent periodic boundary artifacts, without applying any rotational alignment. This translation centering is critical for tilt angle measurements, as rotational alignment would reorient the coordinate frame and invalidate the membrane normal as an absolute reference.

The tilt angle θ was defined as the angle between the helix principal axis vector v and the membrane normal *n* = [0, 0, 1]:

$$\theta =\arccos\left(\frac{v\cdot n}{\left\|v\right\|}\right).$$

To ensure consistent directionality across frames and monomers, the principal axis vector was flipped when its dot product with the membrane normal was negative, restricting all tilt angles to the range 0°–90°. Under this convention, *θ* = 0° indicates a helix oriented parallel to the membrane normal (transmembrane insertion), and *θ* = 90° indicates a helix lying flat within the membrane plane (surface‐parallel adsorption). The tilt from the membrane plane, defined as (90° − θ), is reported to facilitate comparison with experimental NMR and oriented circular dichroism studies, where tilt is conventionally measured from the bilayer surface (Monticelli et al. 2010). Per‐monomer tilt angles were computed for all frames of each replicate and summarized as mean ± standard deviation per monomer. Overall oligomer tilt was reported as the mean across all monomers and all three replicates, with the standard error of the mean (SEM) calculated across replicates.

### 2.11 Statistical Analysis

All quantitative results were reported as mean ± standard error of the mean (SEM) computed across three independent replicate simulations (*n* = 3), treating each replicate trajectory as a single statistically independent observation. For each quantitative metric, a scalar summary value was first computed per replicate by averaging over all production frames (0–200 ns = 201 frames) of that trajectory. The mean and SEM were calculated across the three replicate scalar values, yielding *n* = 3 as the effective sample size for all reported statistics. For time‐series figures, the mean trajectory was computed by averaging frame‐by‐frame values across replicates at each time point, with shaded bands representing ± SEM values. All statistical computations were performed in Python (v3.12.5) using NumPy (v1.26), pandas (v2.3.1), and the scipy. stats module of SciPy (Harris et al. 2020; Reback 2020; Virtanen et al. 2020). Plotting and visualization were performed using Matplotlib, VMD and UCSF ChimeraX 1.10 (Humphrey et al. 1996; Hunter 2007; Pettersen et al. 2021).

## 3 Results and Discussion

### 3.1 Screening of Oligomeric States

**Figure 1:**
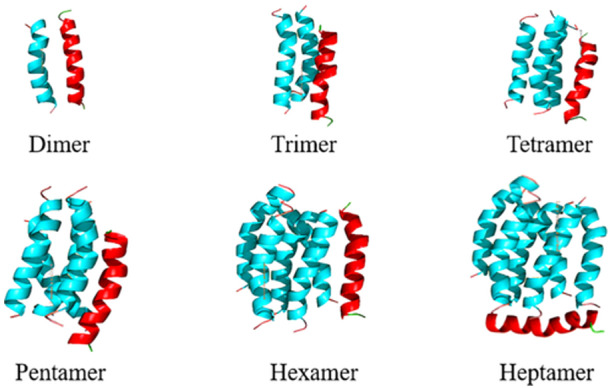
Oligomeric assemblies of Alyteserin‐1c generated by sequential docking using ClusPro 2.0.

Sequential docking of Alyteserin‐1c monomers using ClusPro 2.0 (Kozakov et al. 2017) generated a series of higher‐order oligomeric assemblies ranging from dimers to heptamers (Figure 1). For each oligomeric state, the top‐ranked structure obtained from the Balanced scoring mode was selected for structural assessment. The dimer and trimer displayed stable antiparallel helix–helix packing interactions, a structural arrangement that was retained in the tetrameric and pentameric assemblies. The hexamer adopted a compact bundle‐like architecture in which the helices were symmetrically organized around a central axis, yielding a geometry that is consistent with potential membrane insertion, membrane disruption, and pore‐forming behavior. In contrast, although the heptameric assembly appeared structurally compact, it exhibited noticeable peripheral disorder and reduced packing uniformity and was therefore excluded from further analysis (Figure 1). Considering the combined parameters of docking score, structural compactness, and the outcomes of subsequent rigidity analysis (Section 3.2), the hexameric assembly was identified as the most structurally plausible oligomer for membrane simulation studies. It should be noted that this assembly represents a computationally predicted model derived from rigid‐body docking under vacuum conditions and does not represent an experimentally validated oligomeric state.

### 3.2 Rigidity and Flexibility Analysis of Alyteserin‐1c Oligomers

**Figure 2:**
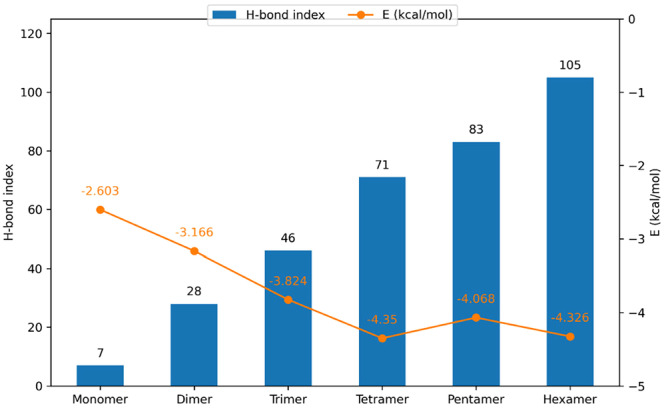
Graphical representation of the rigidity and flexibility profile of Alyteserin‐1c oligomers.

Subsequently, the mechanical stability of Alyteserin‐1c oligomers was evaluated using ProFlex rigidity analysis (Jacobs et al. 2001), with stability assessed on the basis of intramolecular hydrogen bond count (H‐bond index) and the corresponding free energy (ΔE). The analysis demonstrated a clear correlation between oligomerization and structural stabilization. As the oligomeric state progressed from monomer to hexamer, a substantial increase in intramolecular hydrogen bonding was observed, highlighting the importance of these interactions in maintaining peptide stability (Kreutzer et al. 2017). Specifically, the H‐bond index increased from 7 in the monomer to 28 in the dimer and reached a maximum value of 105 in the hexamer (Figure 2). This gradual increase in hydrogen bond formation was accompanied by enhanced energetic stability, with the energy required to disrupt these interactions increasing from −2.603 kcal·mol^−1^ in the monomer to −4.326 kcal·mol^−1^ in the hexamer (Supporting Figure 1). The extensive and densely interconnected hydrogen bond network observed in the hexamer suggested greater resistance to thermal fluctuations, supporting its identification as the most mechanically stable oligomeric assembly.

### 3.3 Molecular Dynamics Simulations of Alyteserin‐1c Hexamer

**Figure 3:**
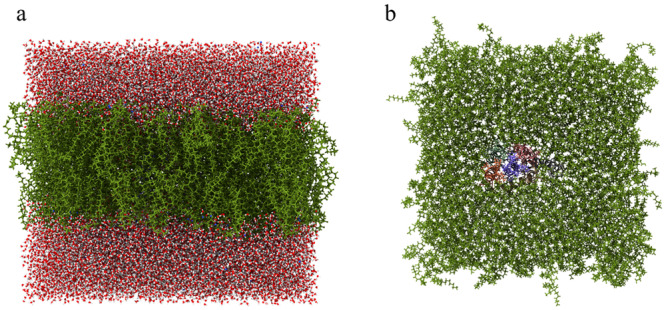
Simulation system of Alyteserin‐1c hexamer in the lipid bilayer. (a) Side view showing the peptide hexamer embedded in the membrane and surrounded by explicit water molecules. (b) Top view illustrating peptide orientation within the bilayer.

To investigate the membrane disruption mechanism of Alyteserin‐1c and to elucidate the molecular basis underlying its selectivity toward Gram‐positive and Gram‐negative bacterial membranes, three independent 200 ns molecular dynamics (MD) simulations were carried out for the hexameric assembly embedded within each membrane model (GP: Gram‐positive inner membrane model; GN: Gram‐negative inner membrane model). The stability of the peptide–membrane systems was confirmed by the convergence of thermodynamic parameters throughout the simulations, with temperature values maintained at 309.999 ± 0.001 K for GN and 309.998 ± 0.001 K for GP systems, while pressure values remained stable at 0.942 ± 0.08 bar and 0.904 ± 0.07 bar, respectively, across all replicates. A representative simulation cell illustrating the hexamer embedded within the membrane environment, together with its top (aerial) view, is shown in Figure 3.

### 3.4 System Energetics and Density

**Figure 4:**
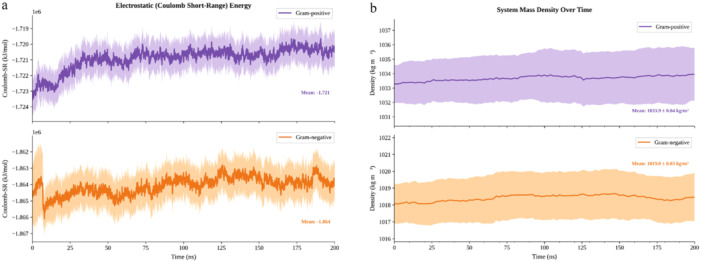
(a) Coulomb short‐range interaction energy of the Alyteserin‐1c hexamer–membrane complex in Gram‐negative and Gram‐positive systems (Mean ± SEM, *n* = 3 replicates). The Gram‐negative system exhibits a larger absolute Coulomb short‐range energy magnitude, reflecting deep structural stabilization. (b) System mass density of Gram‐negative (1019.0 ± 0.03 kg/m^3^) and Gram‐positive (1033.9 ± 0.04 kg/m^3^) membrane simulation systems (Mean ± SEM, *n* = 3 replicates), suggesting the globally denser packing of the saturated PG‐rich Gram‐positive bilayer.

Both membrane systems maintained stable potential energy profiles over the 200 ns simulation period, with average values of −1.292 ± 0.002 × 10^6^ kJ/mol for the GN model and −1.088 ± 0.001 × 10^6^ kJ/mol for the GP model (Supporting Figure S2). The lower absolute potential energy observed for the GN system is primarily attributable to its larger system size and higher degree of hydration compared to the GP system, rather than indicating differences in structural stability. These observations showed that both peptide–membrane systems achieved satisfactory thermodynamic equilibration throughout the simulations. Further, the Coulomb short‐range (Coulomb‐SR) interaction energy averaged −1.864 ± 0.003 × 10^6^ kJ/mol for the GN system and −1.721 ± 0.004 × 10^6^ kJ/mol for the GP system (Figure 4a). In this context, the Coulomb‐SR term represents the cumulative short‐range electrostatic interactions within the entire system, including peptide–lipid, lipid–lipid, peptide–water, and ion–lipid contributions. The larger absolute Coulomb‐SR magnitude observed in the GN system is partly attributable to the greater number of water molecules present in the simulation box. In addition, the high phosphatidylethanolamine (PE) content (75%) of the GN membrane promotes the formation of a stable lipid–lipid electrostatic network through intermolecular NH_3_^+^–PO_4_^−^ hydrogen bonding between neighboring PE headgroups, thereby enhancing the overall electrostatic stabilization of the GN membrane system (Gurtovenko and Vattulainen 2007). Therefore, the total Coulomb‐SR energies are interpreted primarily as indicators of system equilibration and stability, rather than as direct measures of peptide binding affinity toward the membrane.

System density analysis demonstrated the characteristic physical properties of both membrane models. The GP bilayer displayed a higher equilibrium mass density (1033.9 ± 0.04 kg/m^3^) than the GN bilayer (1019.0 ± 0.03 kg/m^3^) (Figure 4b). This increase is consistent with the properties of phosphatidylglycerol‐rich membranes, as phosphatidylglycerol headgroups possess greater molecular mass and distinct hydration behavior relative to phosphatidylethanolamine headgroups (Gurtovenko and Vattulainen 2007). However, the elevated mass density of the GP membrane should not be directly interpreted as evidence of increased mechanical rigidity. In the subsequent sections, the parameters such as acyl chain order and lateral lipid diffusion provide a clear picture of the mechanical properties of each membrane.

### 3.5 Structural Stability of the Hexamer

**Figure 5:**
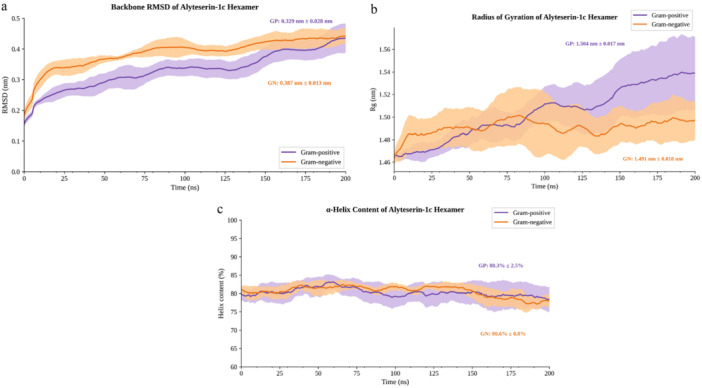
(a) Backbone RMSD time series of the Alyteserin‐1c hexamer in Gram‐negative (orange) and Gram‐positive (purple) membrane models over 200 ns. The stable plateau confirms structural convergence in both systems. Shaded regions represent ± SEM across three independent replicates. (b) Radius of gyration (Rg) of the Alyteserin‐1c hexamer over 200 ns in Gram‐negative and Gram‐positive membrane models. The stable Rg confirms maintained compact hexameric assembly throughout all simulations. Shaded regions represent ± SEM (*n* = 3). (c) α‐Helical content (%) of the Alyteserin‐1c hexamer computed by DSSP over 200 ns in Gram‐negative (orange) and Gram‐positive (purple) membrane systems. Stable helicity (~80%) throughout all replicates confirms preserved amphipathic helix character upon membrane embedding. Shaded regions represent ± SEM (*n* = 3).

The structural stability of the Alyteserin‐1c hexamer was evaluated throughout the simulations using backbone RMSD, radius of gyration (Rg), and α‐helical content analyses. The average RMSD values were 0.387 ± 0.013 nm for the GN system and 0.329 ± 0.020 nm for the GP system. The comparatively higher RMSD observed in the GN membrane suggested increased lateral conformational flexibility of the hexamer within the less densely packed bilayer environment (system density: 1019.0 kg/m^3^). Conversely, the lower RMSD in the GP system is consistent with the more spatially constrained environment imposed by the denser GP membrane (1033.9 kg/m^3^) (Figure 5a). This restriction is likely associated with the tightly packed saturated phosphatidylglycerol‐rich (PG) lipid chains, which limit lateral movement of the peptide assembly. Importantly, both systems exhibited stable RMSD plateaus over the course of the simulations, indicating that the observed differences represent equilibrium conformational behavior rather than structural destabilization or unfolding.

The Rg remained stable throughout the simulations, with average values of 1.491 ± 0.018 nm for the GN system and 1.504 ± 0.012 nm for the GP system (Figure 5b). The marginally higher Rg observed in the GP membrane suggested a more upright and geometrically constrained arrangement of the hexamer within the densely packed bilayer environment. In addition, the α‐helical content of the hexamer was consistently maintained during the simulations, averaging 80.6% ± 0.8% in the Gram‐negative membrane and 80.3% ± 0.5% in the Gram‐positive membrane (Figure 5c). The sustained high helical content indicated that the amphipathic α‐helical architecture of Alyteserin‐1c remains structurally preserved upon membrane insertion, in agreement with the conformational features typically associated with surface‐active carpet and toroidal pore disruption mechanisms (Shai 1999; Subasinghage et al. 2011). Following confirmation of hexamer structural stability, subsequent analyses focused on the molecular determinants governing hexamer cohesion and peptide–lipid interaction networks.

### 3.6 Inter‐Monomer Cohesion and Peptide–Membrane Interactions

#### 3.6.1 Inter‐Monomer Cohesion

Inter‐monomer hydrogen bonds (Gram‐negative: 2.16 ± 0.24; Gram‐positive: 1.71 ± 0.61 per frame) and salt bridges (Gram‐negative: 0.511 ± 0.149; Gram‐positive: 0.761 ± 0.441 per frame) were minimal across all simulation replicates, indicating that electrostatic inter‐monomer interactions do not play a major role in stabilizing the hexameric assembly (Supplementary Figure S3). In contrast, the monomer–monomer (M1–M2) interface contact map demonstrated persistent hydrophobic packing interactions, with residue pairs L10 ↔ L13 (95.2%), V14 ↔ L13 (95.2%), V14 ↔ L10 (90.5%), I17 ↔ L10 (85.7%), and I17 ↔ F6 (81.0%) exhibiting contact occupancies exceeding 80% (Supporting Figure S4) (Huang 2006). Notably, the cationic residues K3, K7, and K15 were absent from the inter‐monomer interface, indicating that these positively charged side chains remain exposed and available for electrostatic interactions with membrane lipid headgroups. This observation is further supported by the near‐complete lipid contact occupancies observed for K7 (> 98%) and K15 (100%) in the subsequent per‐residue lipid interaction analysis.

#### 3.6.2 Peptide–Membrane Interactions

**Figure 6:**
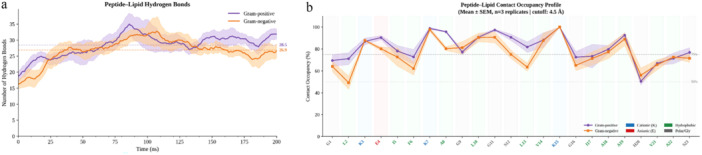
(a) Peptide–lipid hydrogen bonds over 200 ns in Gram‐negative (orange) and Gram‐positive (purple) membrane models. The progressive increase during the first 80 ns reflects a two‐phase insertion process of initial adsorption followed by stable membrane embedding. Shaded regions represent ± SEM across three replicates (*n* = 3). (b) Residue‐level peptide–lipid contact occupancy of the Alyteserin‐1c hexamer averaged across six monomers and three replicates. Residue labels are colored by physicochemical property: blue = cationic (K), red = anionic (E), green = hydrophobic, gray = polar/glycine. Dashed lines indicate HIGH ( ≥ 75%) and MEDIUM (50%–75%) occupancy thresholds.

Peptide‐lipid hydrogen bonding increased progressively during the initial 80 ns of simulation in both membrane systems, rising from approximately 16–20 hydrogen bonds per frame at the beginning of the production run to a stable plateau between 100 and 200 ns. This behavior is indicative of a characteristic two‐stage membrane interaction process, consisting of an initial rapid electrostatically driven surface adsorption phase, followed by a slower stabilization and embedding of the peptide at the lipid–water interface (Schmidt and Wong 2013a). At equilibrium, the average number of peptide–lipid hydrogen bonds reached 26.9 ± 0.7 per frame in the GN system and 28.5 ± 1.5 per frame in the GP system (Figure 6a). The comparatively higher hydrogen‐bonding capacity observed in the GP membrane is consistent with its elevated anionic lipid composition (75% PG), where the greater density of phosphate and hydroxyl‐containing phosphatidylglycerol headgroups promotes the formation of a more extensive hydrogen‐bonding network with the polar residues of the peptide (Talapko et al. 2022).

Peptide‐water hydrogen bonding decreased symmetrically in both membrane systems, declining from approximately 160 hydrogen bonds per frame at the start of the simulations to a stable equilibrium plateau of ~145 hydrogen bonds per frame by 50 ns (Supporting Figure. 5). This reduction in hydration reflects a progressive desolvation process, whereby water molecules are displaced as the amphipathic hexamer inserts its hydrophobic surface into the membrane interior (Schmidt and Wong 2013a). The nearly identical equilibrium hydrogen‐bond counts observed for the Gram‐negative (145.1 ± 4.3 per frame) and Gram‐positive (146.7 ± 4.3 per frame) systems indicated that the overall degree of peptide hydration remains largely conserved between the two membrane environments. These findings suggested that the differential membrane activity of Alyteserin‐1c is primarily governed by lipid‐specific interactions rather than differences in peptide solvation behavior (Supporting Figure S5).

Residue‐level contact occupancy analysis further confirmed the functional importance of the cationic residues in membrane association. Lys7 and Lys15 exhibited near‐continuous interactions with lipid headgroups (K7: > 98%; K15: 100%) in both membrane systems, identifying these residues as the principal electrostatic anchoring sites of the hexamer (Figure 6b). Lys3 also demonstrated high lipid contact occupancy (~87%), whereas His20 showed the lowest occupancy (~43%–64%), consistent with its location at the relatively solvent‐exposed C‐terminal region (Figure 6b). Overall contact occupancies were slightly higher in the GP system, reflecting its greater anionic lipid density (Figure 6b). In contrast, the GN membrane exhibited greater residue‐specific positional variability, particularly for Leu13, which correlates with the increased lateral lipid mobility characteristic of the GN bilayer. Together, the inter‐monomer interaction analysis and residue‐level contact occupancy profiles support a unified structural model in which hydrophobic interactions stabilize the hexameric core, while exposed cationic residues mediate electrostatic anchoring to membrane lipid headgroups (Montis et al. 2024).

### 3.7 Membrane Property Analysis

#### 3.7.1 Membrane Density Profile

**Figure 7:**
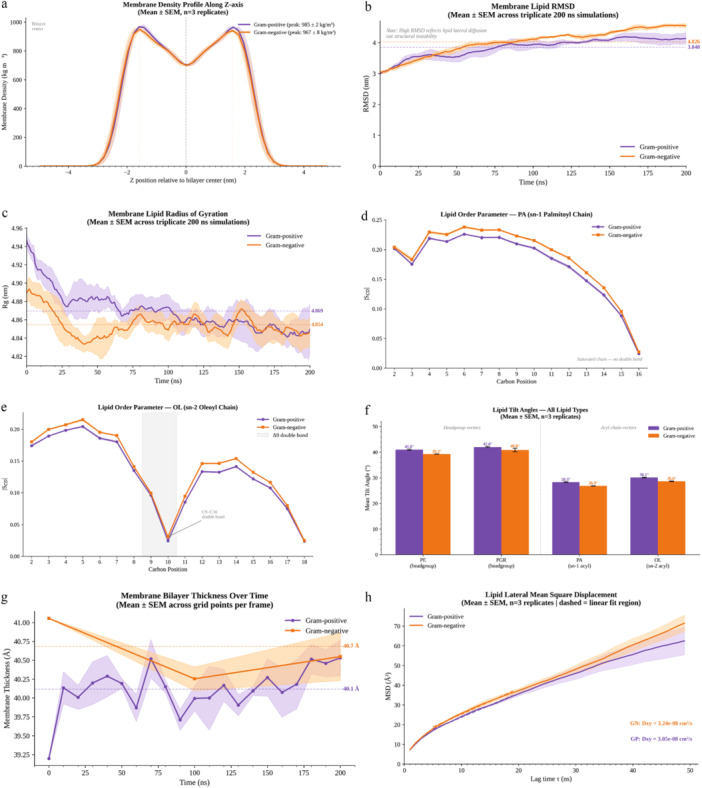
(a) Mass density profile of membrane lipids along the bilayer normal in Gram‐negative and Gram‐positive membrane models (Mean ± SEM, *n* = 3 replicates). (b) Lipid atom RMSD of the Gram‐negative (orange) and Gram‐positive (purple) membrane bilayers over 200 ns. The continuously increasing RMSD reflects normal lateral lipid diffusion rather than structural instability, with the higher final RMSD in GN (4.026 nm) consistent with greater lateral lipid mobility. (c) Radius of gyration (Rg) of membrane lipid atoms in Gram‐negative (orange) and Gram‐positive (purple) systems over 200 ns. The decrease to a stable plateau within 75 ns in both systems indicates membrane compaction and equilibration around the inserted hexamer. Shaded regions represent ± SEM (*n* = 3). (d) Lipid order parameters (S_CD_) of the sn‐1 palmitoyl chain in Gram‐negative (orange) and Gram‐positive (purple) membrane models. The monotonic decrease from C4 to the terminal methyl C16 confirms intact saturated chain organization in both systems. Mean ± SEM, *n* = 3 replicates. (e) Lipid order parameters (S_CD_) of the sn‐2 oleoyl chain in Gram‐negative (orange) and Gram‐positive (purple) membrane models. The characteristic minimum at C10 confirms the cis Δ9 double bond in both systems. Shaded region indicates the double bond position. Mean ± SEM, *n* = 3 replicates. (f) Mean tilt angles of headgroup and acyl chain vectors for all lipid types in Gram‐negative and Gram‐positive membrane models, showing consistently higher tilt in the Gram‐positive system across all lipid classes (Mean ± SEM, *n* = 3 replicates). (g) Membrane bilayer thickness time series computed from MEMBPLUGIN phosphorus atom peak‐to‐peak distances in Gram‐negative (orange) and Gram‐positive (purple) systems over 200 ns. Both systems maintain stable thickness throughout the production run. Shaded regions represent ± SEM (*n *= 3). (h) Lipid lateral mean square displacement (MSD) curves in Gram‐negative (orange) and Gram‐positive (purple) membrane systems computed over the equilibrated trajectory (100–200 ns). Dashed lines indicate the linear fit region used to extract the lateral diffusion coefficient (Dxy) via the Einstein relation. Shaded regions represent ± SEM (*n* = 3).

Mass density profiles calculated along the bilayer normal displayed the characteristic double‐peak distribution in both membrane systems, indicating that bilayer structural integrity was preserved throughout the 200 ns simulations (Figure 7a). The two density maxima located at approximately *Z* = ±1.5 nm from the bilayer center correspond to the lipid headgroup regions of the upper and lower membrane leaflets. Notably, the Gram‐positive membrane showed a higher peak lipid density (985 ± 2 kg/m^3^) than the Gram‐negative membrane (967 ± 8 kg/m^3^) (Figure 7a). This observation suggested tighter lateral packing of the saturated PG‐rich lipids, consistent with the higher equilibrium system density measured for the GP membrane (1033.9 vs. 1019.0 kg/m^3^). Furthermore, the substantially lower inter‐replicate variability observed for the GP profile (SEM ± 2 vs. ± 8 kg/m^3^) supported the greater structural uniformity of the globally denser Gram‐positive bilayer. The central trough at *Z* = 0 ( ~ 710 kg/m^3^ in both systems) corresponds to the disordered terminal methyl groups and remained preserved throughout all simulations, indicating that no continuous aqueous channel formed across the hydrophobic core of either bilayer. This observation is further supported by the pore radius analysis presented in Section 3.9.

#### 3.7.2 Membrane Structural Stability

Lipid RMSD increased progressively throughout the 200 ns simulations in both membrane systems, reflecting normal lateral lipid diffusion rather than structural instability (Wong‐Ekkabut and Karttunen 2016). The Gram‐negative membrane reached a higher final RMSD value (4.026 nm) than the Gram‐positive membrane (3.848 nm), indicating greater lateral lipid mobility in the Gram‐negative bilayer (system density 1019.0 kg/m^3^) relative to the more densely packed Gram‐positive membrane (1033.9 kg/m^3^) (Figure 7b). The Rg of the lipid bilayer decreased from approximately 4.90 nm to a stable plateau of ~4.85–4.87 nm within the first 75 ns in both systems, suggesting membrane compaction and equilibration around the embedded hexamer. At equilibrium, the Gram‐positive membrane retained a marginally higher Rg value (4.869 nm in GP vs. 4.854 nm in GN), indicative of a slightly more extended spatial organization despite the overall higher density of the Gram‐positive bilayer (Figure 7c).

#### 3.7.3 Lipid Order Parameters

Lipid acyl chain order parameters (SCD) were calculated for the sn‐1 palmitoyl (PA) and sn‐2 oleoyl (OL) chains to evaluate the influence of hexamer insertion on membrane fluidity and lipid packing. For the PA (sn‐1) chain, the Gram‐negative membrane displayed consistently higher SCD values across the plateau region (C4–C8; GN ~ 0.231, GP ~ 0.220), indicating a greater degree of acyl chain ordering in the Gram‐negative system (Figure 7d). In both membranes, SCD values decreased progressively from the glycerol‐proximal region toward the terminal methyl group, consistent with the expected organization of saturated lipid chains. The slight dip observed at C3 arises from the glycerol backbone geometry defined in the AMBER LIPID17 force field rather than from physical disorder within the membrane (Figure 7d) (Gould et al. 2018). For the OL (sn‐2) chain, both systems exhibited a characteristic SCD minimum at C10 (GN: 0.031; GP: 0.025), confirming the presence of the cis‐Δ9 double bond and validating the LIPID17 parameterization of the oleoyl chain. Across the plateau region (C2–C7), SCD values remained consistently higher in the GN membrane (GN ~ 0.197, GP ~ 0.186) (Figure 7e). The elevated SCD values in the GN system likely reflect peptide‐induced local ordering or compression rather than an intrinsically more rigid membrane. Specifically, the strong electrostatic anchoring of Lys7 and Lys15 to Gram‐negative lipid headgroups (> 98% and 100% contact occupancy, respectively) effectively restrains neighboring lipid chains and limits their conformational flexibility near the peptide insertion site (Kandasamy and Larson 2006). This interpretation is further supported by the observation that Gram‐negative lipids exhibit greater overall mobility, as indicated by higher lateral RMSD and lipid displacement (Dxy), while simultaneously showing increased local ordering adjacent to the peptide. These findings are consistent with DSC studies by Aragón‐Muriel et al (Aragón‐Muriel et al. 2019)., which demonstrated that Alyteserin‐1c perturbs membrane phase behavior and fluidity in PC/PG model membranes. Collectively, these results support a carpet‐mode interfacial membrane disruption mechanism rather than the formation of stable transmembrane pores.

#### 3.7.4 Lipid Tilt Angles

Lipid tilt angle analysis demonstrated consistently higher tilt values in the Gram‐positive membrane for all lipid types examined. Headgroup vector tilt angles were measured at 41.0° ± 0.3° (PE) and 42.0° ± 0.5° (PGR) in the Gram‐positive system, compared with 39.3° ± 0.4° (PE) and 40.8° ± 0.4° (PGR) in the Gram‐negative membrane (Figure 7f). Similarly, acyl chain tilt angles were higher in the Gram‐positive bilayer, with values of 28.3° ± 0.3° (PA) and 30.1° ± 0.3° (OL), whereas the corresponding values in the Gram‐negative system were 26.9° ± 0.2° and 28.6° ± 0.1°, respectively (Figure 7f). The consistently elevated tilt angles observed in the Gram‐positive membrane likely arise from the tighter lateral packing of saturated PG‐rich lipid chains, which tend to adopt more tilted conformations as an efficient space‐filling arrangement in densely packed bilayers (Das et al. 2025; Seelig 1977). Together with the higher membrane mass density and lower lateral lipid mobility, these results indicated that the Gram‐positive bilayer represents the more structurally constrained membrane environment. In contrast, the lower tilt angles in the Gram‐negative bilayer suggested more vertically oriented acyl chains, consistent with the cone‐shaped geometry of PE lipids and the enhanced local SCD perturbations observed near the peptide insertion region.

#### 3.7.5 Membrane Thickness

Bilayer thickness was determined using MEMBPLUGIN 1.1 in VMD by calculating the peak‐to‐peak distance between phosphorus atoms (P31) of the upper and lower membrane leaflets. The Gram‐negative membrane displayed a slightly greater average thickness (40.69 ± 0.04 Å) than the Gram‐positive membrane (40.12° ± 0.04 Å) (Figure 7g). In contrast, the Gram‐positive bilayer exhibited more pronounced frame‐to‐frame thickness fluctuations over the 200 ns simulation period, reflecting membrane equilibration around the inserted hexamer from an initial thickness of approximately 39.2 Å (Figure 7g). Both membrane systems reached stable equilibrium thickness values within the first 50 ns. The Gram‐negative bilayer equilibrated from a comparatively higher initial thickness, whereas the Gram‐positive membrane evolved from a thinner initial state, consistent with hexamer‐induced bilayer reorganization. Throughout the simulations, both systems maintained thickness values within the expected range for PE/PG‐type bilayers. Comparable behavior has previously been reported for amphipathic α‐helical peptides, where membrane thinning and dynamic thickness fluctuations are associated with surface adsorption and interfacial membrane disruption rather than the formation of stable transmembrane pores (Schmidt and Wong 2013b).

#### 3.7.6 Lipid Lateral Diffusion

Lipid lateral diffusion coefficients (Dxy), determined from the slope of the mean square displacement using the Einstein relation, were calculated as 3.24 ± 0.08 × 10^−8^ cm^2^/s for the Gram‐negative membrane and 3.05 ± 0.06 × 10^−8^ cm^2^/s for the Gram‐positive membrane (Figure 7h). Both values fall within the expected range for peptide‐perturbed PE/PG bilayers and are markedly lower than the reported diffusion coefficient for peptide‐free POPE/POPG membranes (~8.5 × 10^−7^ cm^2^/s), indicating that hexamer insertion reduces lipid lateral mobility in both systems through membrane anchoring and increased acyl chain ordering (Shahane et al. 2019; Goose and Sansom 2013; Park et al. 2024). The consistently higher Dxy values observed in the Gram‐negative membrane across all three replicates further support the higher lipid RMSD measured for this system (4.026 vs 3.848 nm), independently confirming greater overall lateral lipid mobility in the Gram‐negative bilayer. Although the GN/GP diffusion ratio is relatively modest (1.06×; 3.24/3.05), it contributes to a consistent multi‐parameter trend in which system density, peak lipid density, RMSD, and Dxy collectively indicate enhanced global lateral mobility in the Gram‐negative membrane. At the same time, the elevated local SCD values near the peptide insertion site displayed stronger peptide‐induced local perturbation within the Gram‐negative bilayer (Makowski et al. 2025).

### 3.8 Helix Tilt Analysis

**Figure 8:**
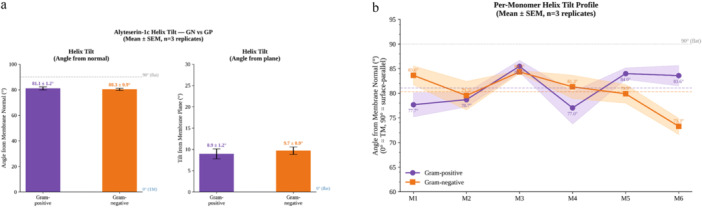
(a) Helix tilt analysis of Alyteserin‐1c hexamer in Gram‐positive and Gram‐negative membranes showing near‐parallel membrane surface orientation consistent with carpet/toroidal AMP disruption mechanisms. (b) Per‐monomer helix tilt profile of Alyteserin‐1c hexamer showing greater tilt asymmetry in the Gram‐negative membrane, with deeper insertion of M6, compared to the more uniform surface‐parallel orientation in the Gram‐positive membrane.

Per‐monomer helix tilt analysis showed that all six Alyteserin‐1c monomers maintained a predominantly surface‐parallel orientation in both membrane systems throughout the 200 ns simulations. The mean tilt angles relative to the membrane normal were 80.3° ± 0.9° in the Gram‐negative membrane and 81.1° ± 1.2° in the Gram‐positive membrane, corresponding to orientations of approximately 9.7° and 8.9° relative to the membrane plane, respectively (Figure 8a). This near‐parallel adsorption geometry is a hallmark of carpet and toroidal antimicrobial peptide disruption mechanisms and is incompatible with the transmembrane insertion characteristic of the classical barrel‐stave pore model, which would require tilt angles approaching 0° relative to the membrane normal (Huang 2000; Bechinger 1997) (Figure 8a). The observed surface‐parallel orientation is also consistent with the amphipathic α‐helical architecture of Alyteserin‐1c, in which hydrophobic and cationic residues are spatially segregated on opposite faces of the helix, thereby favoring interfacial membrane adsorption (Subasinghage et al. 2011).

Per‐monomer analysis demonstrated an asymmetric tilt distribution within the hexamer in the Gram‐negative membrane, where the sixth peptide monomer (M6) displayed the lowest tilt angle (73.3° relative to the membrane normal), suggesting comparatively deeper insertion of this monomer, likely driven by electrostatic interactions with anionic PGR lipids (Figure 8b). In contrast, the Gram‐positive system exhibited a more uniform tilt distribution (77.0°–84.5°), consistent with the more constrained and symmetric hexamer geometry imposed by the globally denser Gram‐positive bilayer (Figure 8b). The greater monomer‐to‐monomer tilt variability observed in the Gram‐negative membrane relative to the Gram‐positive system reflects the broader trend of enhanced hexamer conformational flexibility within the more laterally mobile Gram‐negative bilayer, as also reflected in the RMSD, contact occupancy, membrane thickness, and density analyses (Aragón‐Muriel et al. 2019). Furthermore, the predominantly surface‐parallel helix orientation, together with the absence of transmembrane insertion, is corroborated by the pore radius and water dynamics analyses presented in the following section.

### 3.9 Pore Radius and Water Dynamics

#### 3.9.1 Pore Radius

**Figure 9:**
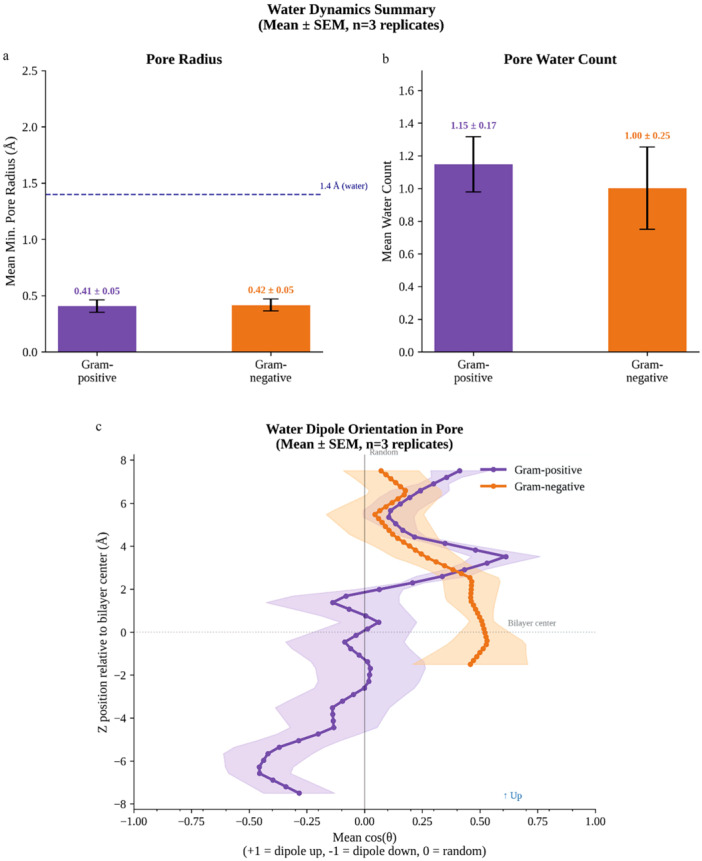
Pore geometry and water dynamics of the Alyteserin‐1c hexamer. (a) Mean minimum pore radius from HOLE2 analysis; dashed line indicates the 1.4 Å water‐permeable threshold. (b) Mean pore water count within a 5 Å radius cylinder centered on the hexamer pore axis. (c) Water dipole orientation profile (mean cos θ vs. Z position); the sign flip in the Gram‐positive system indicates transient single‐file water threading, while the Gram‐negative system shows upper‐leaflet restricted disruption. Mean ± SEM, *n* = 3 replicates.

HOLE2 pore radius analysis demonstrated that neither membrane system formed a stable water‐permeable transmembrane pore during the 200 ns simulations (Supporting Figure. 6). The mean minimum pore radii were 0.42 ± 0.05 Å in the Gram‐negative system and 0.41 ± 0.05 Å in the Gram‐positive system, with both values remaining well below the ~1.4 Å threshold required for the passage of a single water molecule (Figure 9a) (Smart et al. 1996). These pore radius values were consistently reproduced across all three simulation replicates in both membrane systems, indicating that the absence of a stable pore represents an inherent property of the Alyteserin‐1c hexameric assembly at this timescale rather than a stochastic simulation artifact. Such observations are consistent with pre‐pore or interfacial disruption states reported for other short AMPs at sub‐saturation concentrations, where stable pore formation generally requires longer simulation times or higher peptide‐to‐lipid ratios than those investigated in the present study (Lee et al. 2016).

#### 3.9.2 Pore Water Count

Mean pore water occupancy was approximately one molecule per frame in both membrane systems (GN: 1.00 ± 0.25; GP: 1.15 ± 0.17 molecules per frame), indicating the absence of sustained transmembrane water flux in either bilayer (Figure 9b). The greater variability observed in the Gram‐negative membrane (SEM ± 0.25 vs. ± 0.17 in GP) suggests more stochastic and transient water entry events, consistent with the higher lateral lipid mobility of the Gram‐negative bilayer. In contrast, the slightly higher yet more uniform water occupancy in the Gram‐positive system likely reflects the more geometrically constrained hexamer arrangement imposed by the denser Gram‐positive membrane environment. Overall, water occupancy remained negligible in both systems throughout the simulations (Supporting Figure S7).

#### 3.9.3 Water Dipole Orientation

Water dipole orientation analysis revealed the most mechanistically significant distinction between the two membrane systems. In the Gram‐positive membrane, water molecules displayed an oriented dipole alignment spanning across the entire bilayer thickness (*Z* = −8 to +8 Å relative to the bilayer center), with cos(θ) values of approximately −0.5 in the lower leaflet and +0.5 in the upper leaflet. The reversal in dipole orientation at the bilayer center is consistent with transient single‐file water threading through the hexamer interior (Figure 9c). Although this behavior does not indicate the presence of a stable open pore, it suggests that the hexamer within the Gram‐positive bilayer can transiently adopt a more symmetric configuration capable of supporting oriented water organization, a feature associated with early‐stage membrane disruption processes. In contrast, the Gram‐negative system exhibited water dipole ordering confined primarily to the upper leaflet interface (*Z* = 0 to +8 Å), indicating localized interfacial membrane perturbation without complete transmembrane water permeation (Figure 9c).

We hypothesize that this distinction arises from the differing physical constraints imposed by the two membrane environments. The globally denser Gram‐positive bilayer likely restricts the hexamer into a more upright and symmetric configuration that transiently facilitates oriented water threading, whereas the more laterally mobile Gram‐negative bilayer allows greater positional flexibility of the hexamer, thereby disrupting the continuity of transient water wires. Collectively, the pore radius, water occupancy, and dipole orientation analyses support the presence of a pre‐pore interfacial disruption state in both membrane systems rather than the formation of stable transmembrane pores. In the Gram‐negative membrane, selectivity appears to arise from stronger electrostatic anchoring of the peptide and more effective local lipid perturbation. In contrast, the globally denser packing of the Gram‐positive bilayer likely necessitates higher peptide concentrations to achieve comparable levels of membrane disruption. This interpretation is consistent with the four‐fold difference in MIC reported experimentally by Conlon et al. (2009) (Conlon et al. 2009).

## 4 Conclusion

This study provides a comprehensive all‐atom molecular dynamics investigation of the Alyteserin‐1c hexamer in biomimetic Gram‐positive and Gram‐negative inner membrane models using three independent 200 ns simulations for each system (total simulation time: 1.2 μs) to elucidate the membrane disruption mechanism and molecular basis of lipid selectivity of this cationic antimicrobial peptide. Among the oligomeric assemblies examined, the hexamer was identified as the most mechanically stable and geometrically compact structure based on ProFlex rigidity analysis. Following membrane insertion, the hexamer retained structural stability throughout all simulations, as evidenced by stable backbone RMSD values, preservation of α‐helical content (~80%), and maintenance of a compact radius of gyration in both membrane environments. Inter‐monomer stabilization was primarily mediated by hydrophobic packing interactions involving residues L10, L13, V14, I17, and F6 at the oligomer interface, whereas the cationic residues K7 and K15 remained exposed to the lipid environment and functioned as the principal electrostatic anchoring sites, exhibiting contact occupancies greater than 98% and 100%, respectively. The Gram‐negative membrane displayed greater global lateral lipid mobility, reflected by higher lipid RMSD and Dxy values, lower overall membrane density, and stronger local peptide‐induced lipid chain ordering near the insertion region, as indicated by elevated SCD values. Together with the stronger total Coulomb‐SR electrostatic interactions observed in the Gram‐negative system, these findings provide a molecular‐level explanation consistent with the difference in MIC reported experimentally by Conlon et al. (2009) (Conlon et al. 2009). Helix tilt analysis revealed that the peptide monomers adopted a predominantly surface‐parallel orientation (~80° relative to the membrane normal) in both membrane systems. Consistent with this geometry, HOLE2 pore radius analysis demonstrated the absence of stable transmembrane pore formation, with mean minimum pore radii of 0.42 ± 0.05 Å in the Gram‐negative membrane and 0.41 ± 0.05 Å in the Gram‐positive membrane. These observations collectively support a carpet‐type or toroidal interfacial membrane disruption mechanism rather than classical barrel‐stave pore formation. Water dipole orientation analysis revealed the clearest mechanistic distinction between the two membrane systems. In the Gram‐positive membrane, the denser bilayer environment constrained the hexamer into a more upright and symmetric configuration that transiently supported single‐file water threading across the bilayer thickness. In contrast, the Gram‐negative membrane exhibited dipole ordering restricted primarily to the upper leaflet interface, consistent with a surface‐localized disruption mechanism that can be achieved more efficiently at lower peptide concentrations. Overall, the molecular‐level insights obtained in this study into the electrostatic and biophysical determinants of Gram‐negative membrane selectivity provide a framework that may aid the future rational design of membrane‐targeting antimicrobial peptides with enhanced potency and selectivity.

## 5 Limitations

Certain important limitations of this study should be acknowledged. The hexameric assembly was generated by rigid‐body docking in vacuum using ClusPro 2.0 and does not represent a membrane‐assembled oligomeric state; global monomer rearrangement would require more time‐scale simulations. The membrane models represent simplified biomimetic bilayers and do not include the full complexity of biological bacterial envelopes, such as LPS in the Gram‐negative outer membrane or peptidoglycan.

Future studies employing coarse‐grained molecular dynamics, umbrella sampling, or spontaneous self‐assembly of peptide simulations, together with experimental validation such as liposome leakage assays and cross‐linking mass spectrometry, would strengthen the mechanistic conclusions presented here.

## Author Contributions

**Akshay Sasidharan:** conceptualization, optimization, analysis, data curation, visualization, writing – original draft. **Rajasekaran Ramalingam:** conceptualization, supervision, resources, writing – review and editing, project administration.

## Funding

The authors have nothing to report.

## Ethics Statement

The authors have nothing to report.

## Conflicts of Interest

The authors declare no conflicts of interest.

## Declaration of Generative AI and AI‐Assisted Technologies in the Writing Process

The author used Claude (Anthropic) to help debug and correct Python scripts for MDAnalysis trajectory analysis, Matplotlib visualization, and statistical calculations. ProWritingAid and ChatGPT (OpenAI) to improve grammar and readability of the manuscript. All the script and data analysis were independently verified and approved by the authors, who take full responsibility for the accuracy and integrity of the final published work.

## Supporting information

Supporting File

## Data Availability

The datasets generated and analyzed during this study, including trajectory files, analysis scripts, and processed data, are available upon reasonable request. Additional supporting information is included in the Supplementary Information Section.
