# Supplementary material for: Exploring the Mechanism of Alyteserin‐1c in Gram‐Positive and Gram‐Negative Bacterial Membrane Models Using a Computational Approach
Source: Microbiologyopen. 2026 Jun 10;15(3):e70327. doi: 10.1002/mbo3.70327 (PMC13253612; doi:10.1002/mbo3.70327)
Supplement: Supplementary file 1 — Supporting File [file MBO3-15-e70327-s001.docx]

**Supplementary Information**


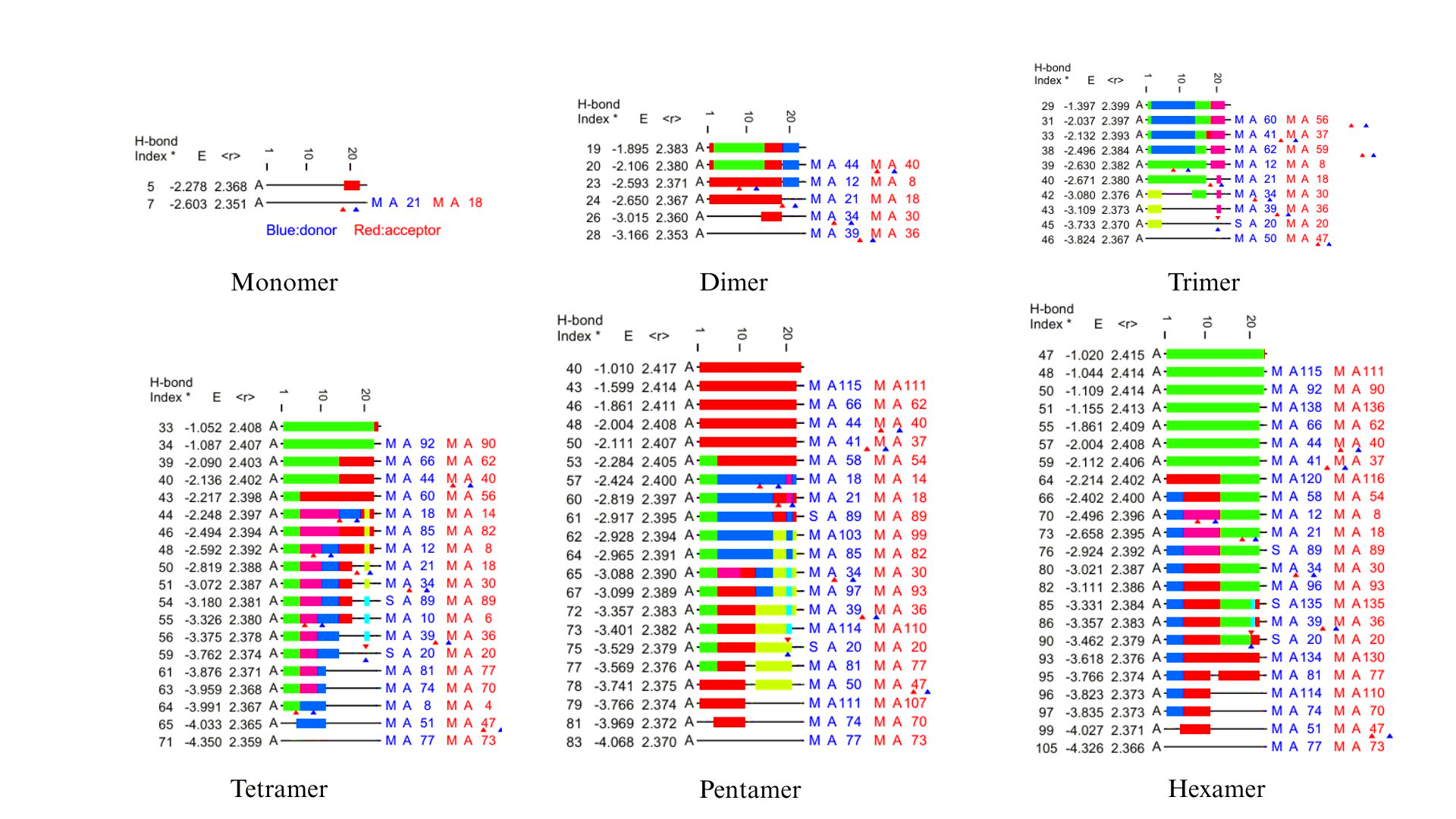


**Supplementary Figure.1**: Rigidity and Flexibility profile of Alyteserin-1c oligomers obtained from ProFlex analysis, highlighting the progressive increase in hydrogen bonding and stabilization from monomer to hexamer.


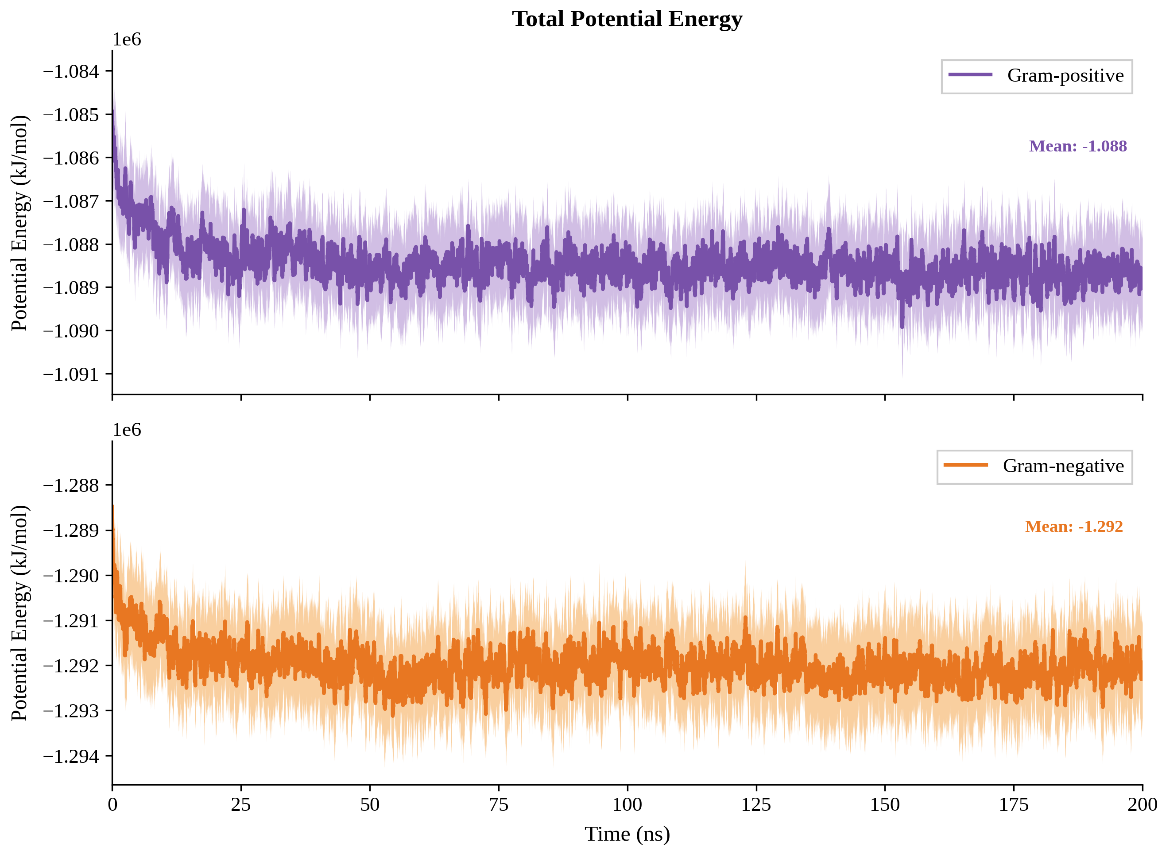


**Supplementary Figure 2.** Total potential energy time series of Gram-positive (upper panel) and Gram-negative (lower panel) membrane systems over 200 ns, confirming thermodynamic stability throughout all production simulations. The difference in absolute values reflects the larger atom count of the Gram-negative simulation box. Shaded regions represent ± SEM (n=3).


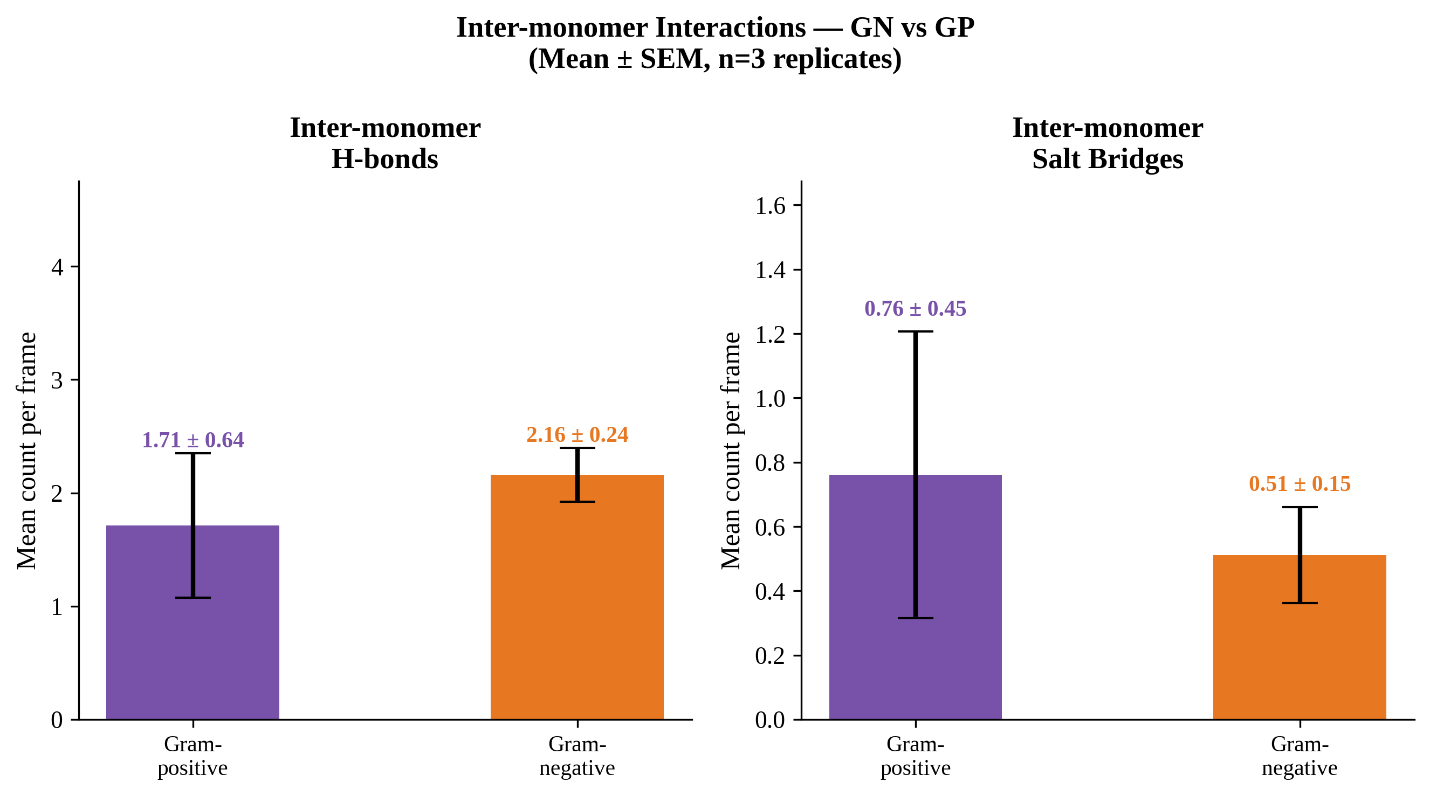


**Supplementary Figure. 3.** Time series of inter-monomer (a) hydrogen bonds and (b) salt bridges within the Alyteserin-1c hexamer in Gram-negative (orange) and Gram-positive (purple) membrane models over 200 ns. Negligible values in both systems confirm that hexamer cohesion is maintained by hydrophobic packing rather than electrostatic inter-monomer contacts. Shaded regions represent ± SEM (n=3).


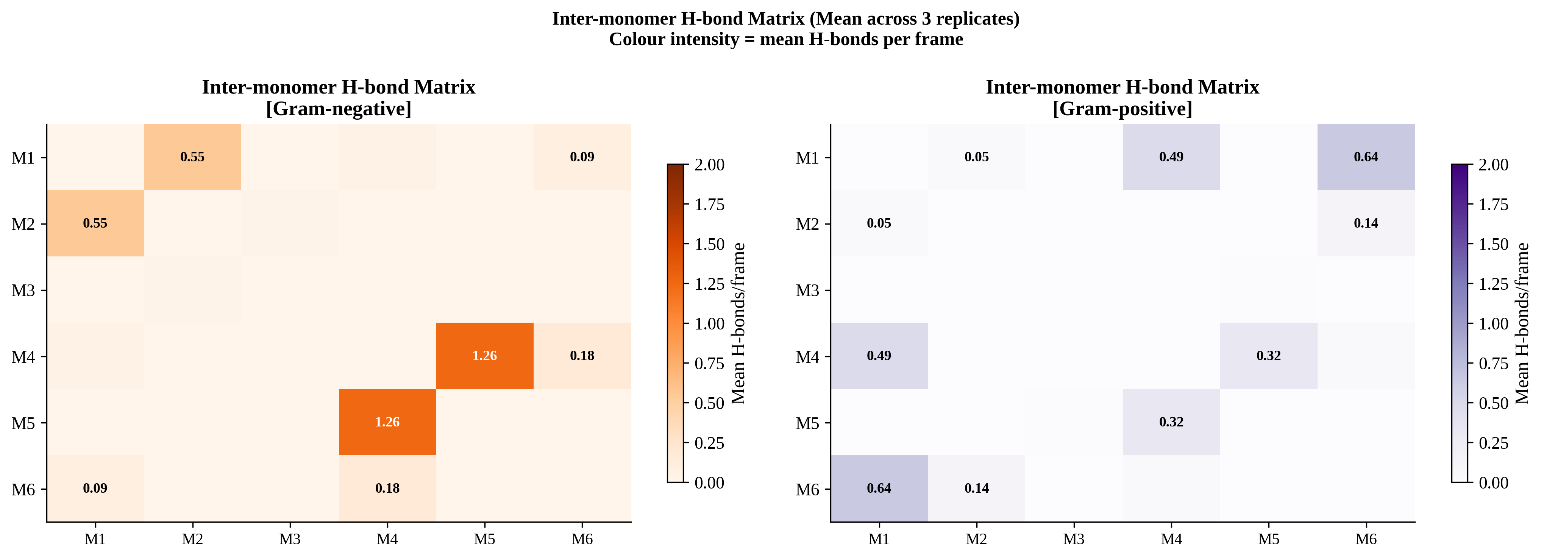


**Supplementary Figure. 4.** Inter-monomer hydrogen bond frequencies for all monomer pairs showing detectable interactions in Gram-negative (orange) and Gram-positive (purple) membrane systems (Mean ± SEM, n=3). The M4–M5 pair contributes the most in GN and M1–M6 in GP, though values remain negligible in both systems.

**
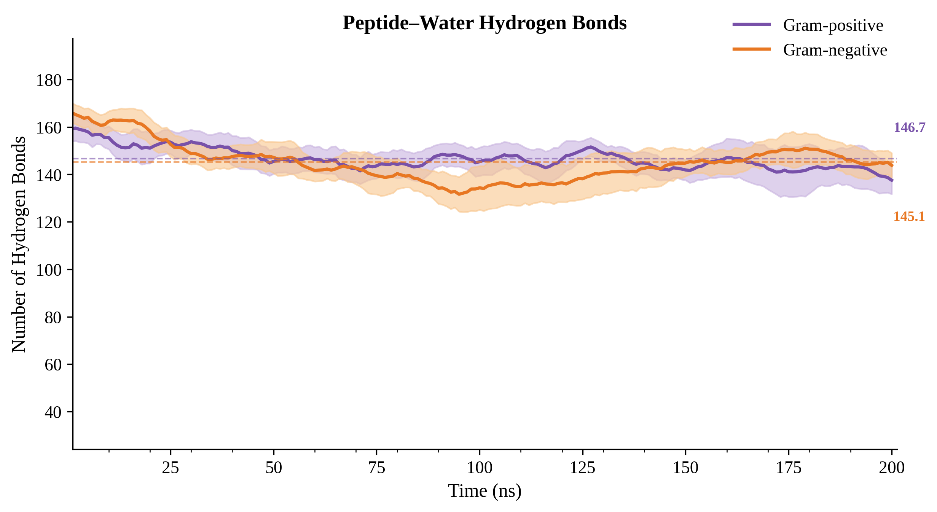
**

**Supplementary Figure. 5.** Peptide–water hydrogen bonds over 200 ns in Gram-negative (orange) and Gram-positive (purple) membrane models. The decreasing trend during the first 50 ns reflects progressive displacement of surface water upon membrane embedding, reaching an identical equilibrium (~145 bonds/frame) in both systems. Shaded regions represent ± SEM (n=3).

**
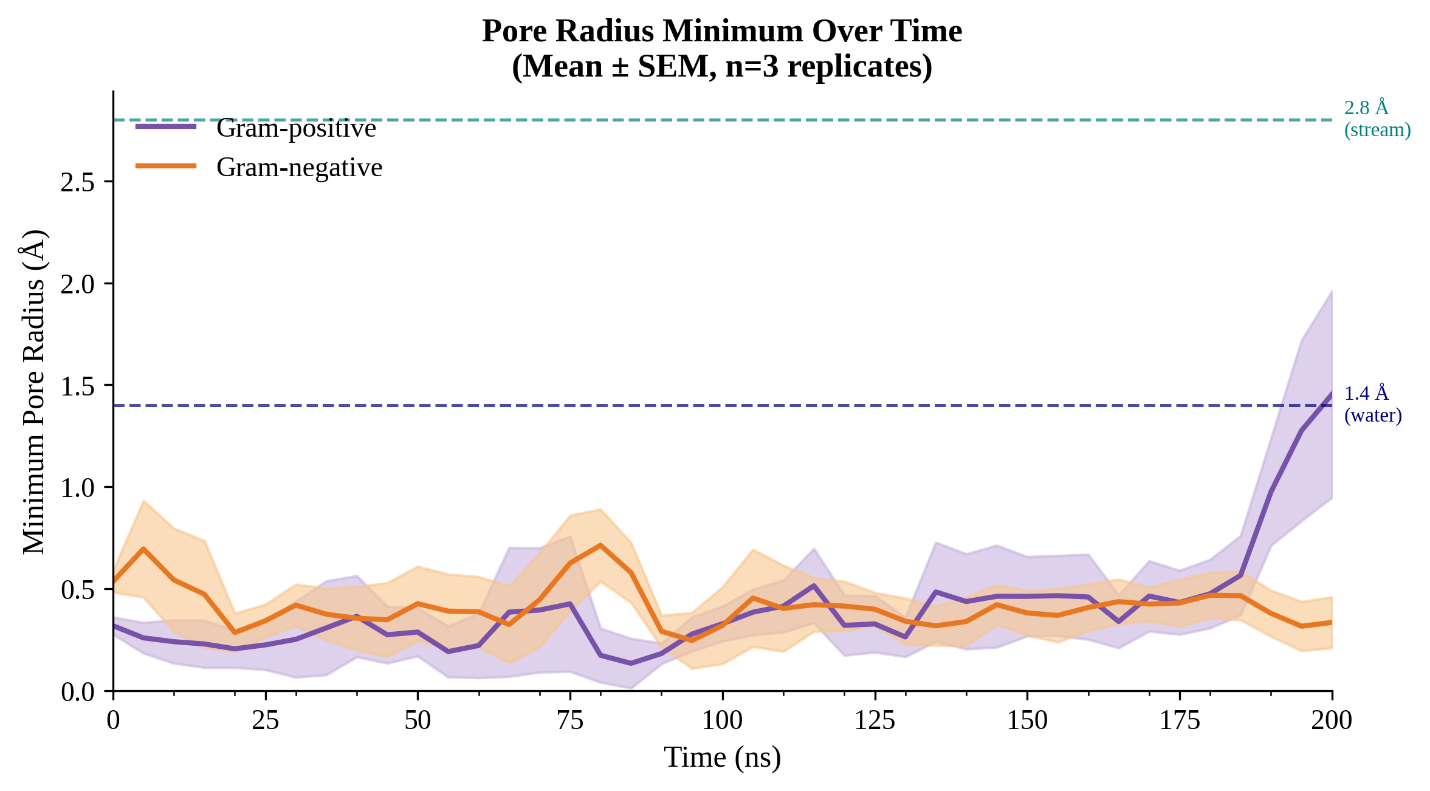
**

**Supplementary Figure. 6.** Minimum pore radius over time from HOLE2 analysis of the Alyteserin-1c hexamer in Gram-negative (orange) and Gram-positive (purple) membrane models. Values consistently below the 1.4 Å water-permeable threshold (dashed line) confirm the absence of a stable transmembrane pore in both systems throughout 200 ns. Shaded regions represent ± SEM (n=3).


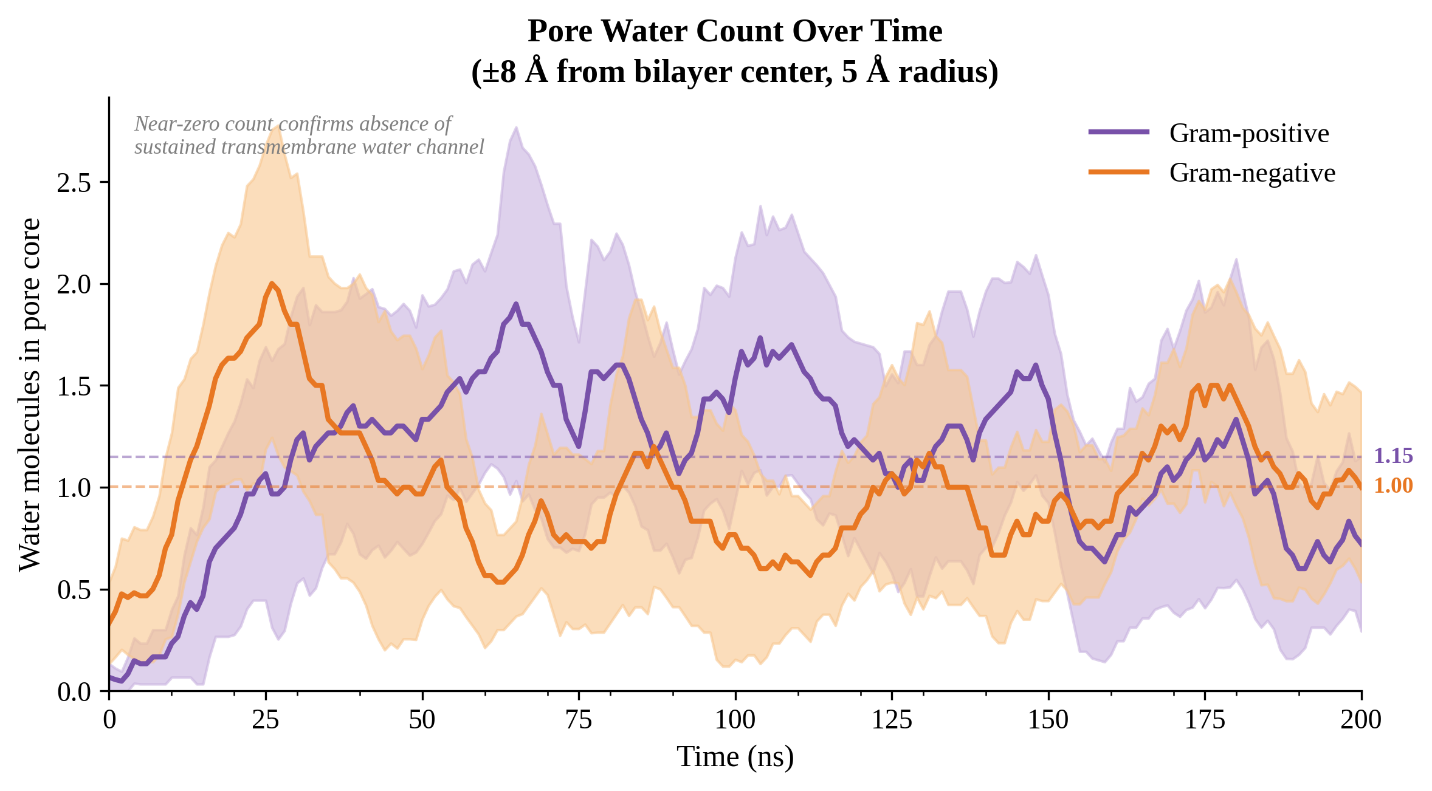


**Supplementary Figure. 7**. Pore water count over 200 ns in Gram-negative (orange) and Gram-positive (purple) membrane models. Mean occupancy of approximately one water molecule per frame in both systems confirms the absence of sustained transmembrane water flux. Shaded regions represent ± SEM (n=3).
